# Supplementary material for: Integrated Metabolomics and Morpho-Biochemical Analyses Reveal a Better Performance of Azospirillum brasilense over Plant-Derived Biostimulants in Counteracting Salt Stress in Tomato
Source: Int J Mol Sci. 2022 Nov 17;23(22):14216. doi: 10.3390/ijms232214216 (PMC9698407; doi:10.3390/ijms232214216)
Supplement: Supplementary file 1 [file ijms-23-14216-s001.zip › Supplementary Figure S1.pdf]

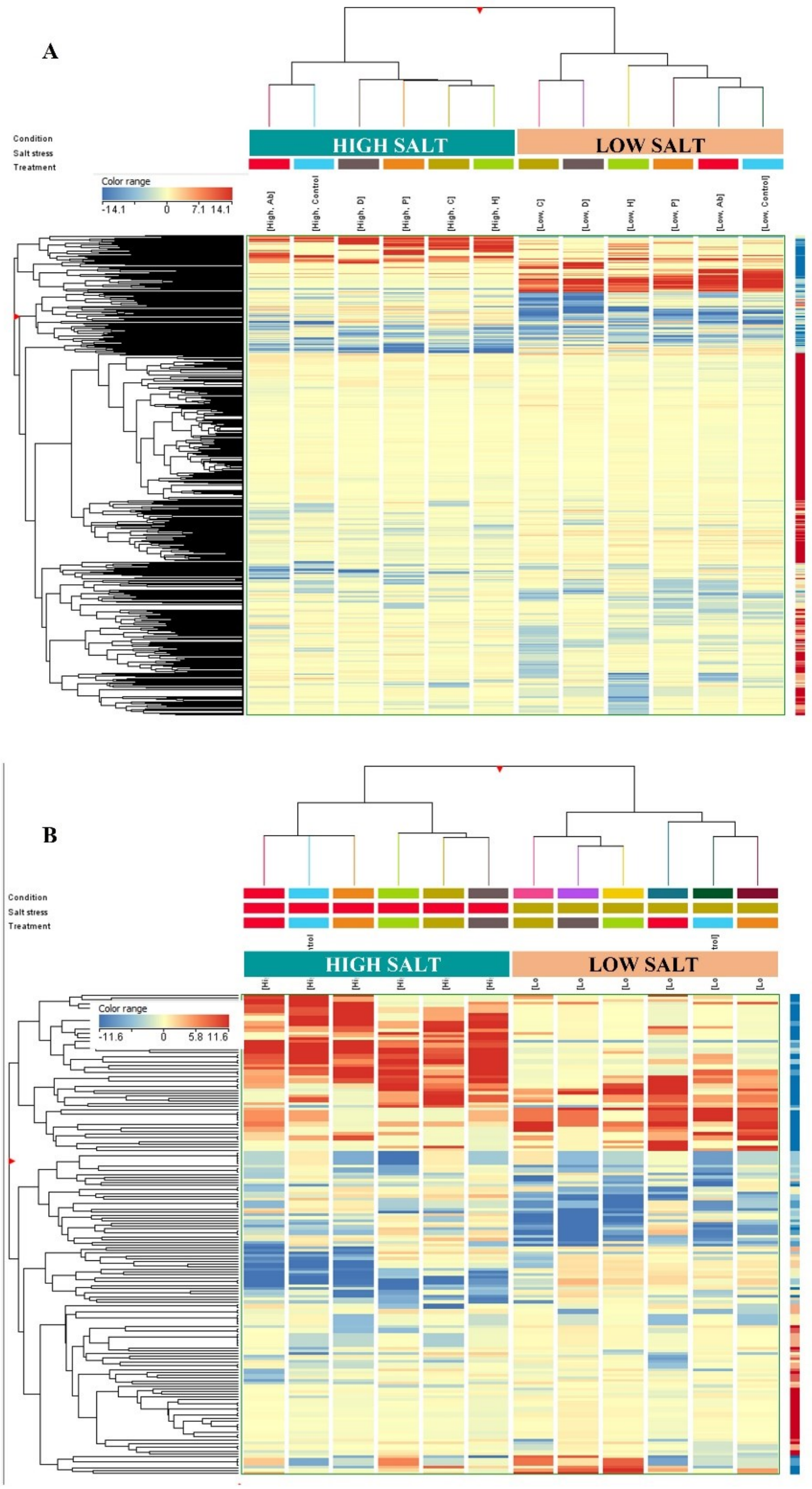

Supplementary Figure S1. Unsupervised hierarchical cluster analysis carried out from UHPLC-ESI/QTOF-MS metabolomic analysis of leaves (A) and root exudates (B) of tomato plants under low salt or high salt conditions, treated with either protein hydrolysates-based biostimulants (PHs), or with PGPR *A. brasilense*. The fold-change based heat map was used to build hierarchical clusters (linkage rule: Ward; distance: Euclidean).
