# Supplementary material for: Integrated Metabolomics and Morpho-Biochemical Analyses Reveal a Better Performance of Azospirillum brasilense over Plant-Derived Biostimulants in Counteracting Salt Stress in Tomato
Source: Int J Mol Sci. 2022 Nov 17;23(22):14216. doi: 10.3390/ijms232214216 (PMC9698407; doi:10.3390/ijms232214216)
Supplement: Supplementary file 1 [file ijms-23-14216-s001.zip › Supplementary Figure S2.pdf]

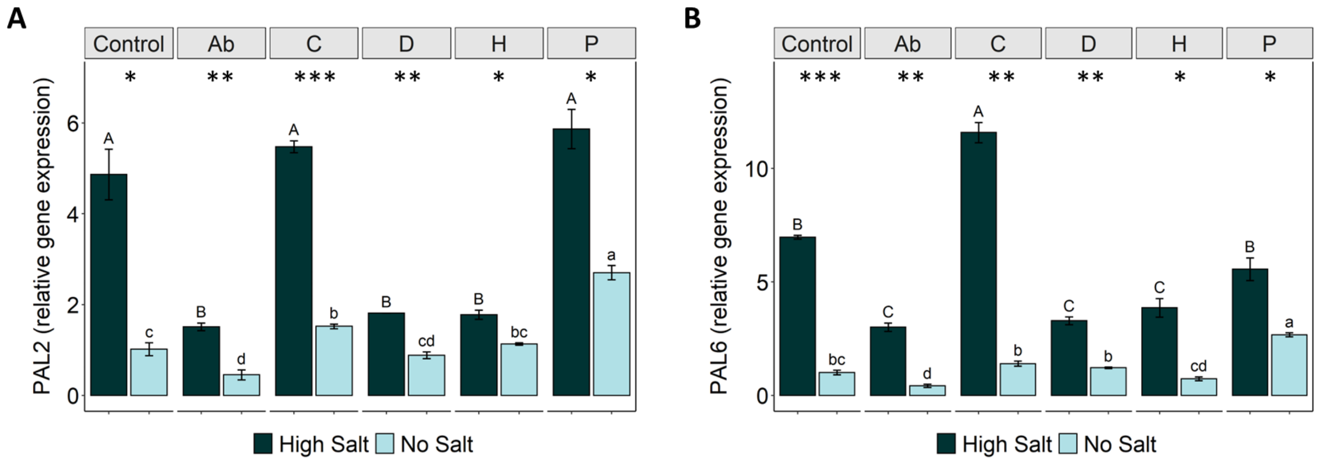

Supplementary Figure S2. Gene expression analysis of (**A**) *PAL2* and (**B**) *PAL6* in the leaves of tomato grown hydroponically under two salinity levels and six biostimulant treatments. The expression level of each gene was normalized to the expression level of the elongation factor isoform 1-alpha (EF-1a). The relative expression ratios were calculated using the untreated control under no salt conditions as a calibrator sample. Values are means ± SE; n = 3. Uppercase letters compare treatments under high salt and lowercase letters compare treatments under no salt. Equal letters correspond to average values that do not differ according to Tukey's test ($p < 0.05$). Asterisks are present when there is difference in the same treatment between high salt and low salt according to t-test (*$p < 0.05$, **$p < 0.01$, ***$p < 0.001$).
