# Supplementary figures and images for: Integrated Metabolomics and Morpho-Biochemical Analyses Reveal a Better Performance of Azospirillum brasilense over Plant-Derived Biostimulants in Counteracting Salt Stress in Tomato
Source: Int J Mol Sci. 2022 Nov 17;23(22):14216. doi: 10.3390/ijms232214216 (PMC9698407; doi:10.3390/ijms232214216)

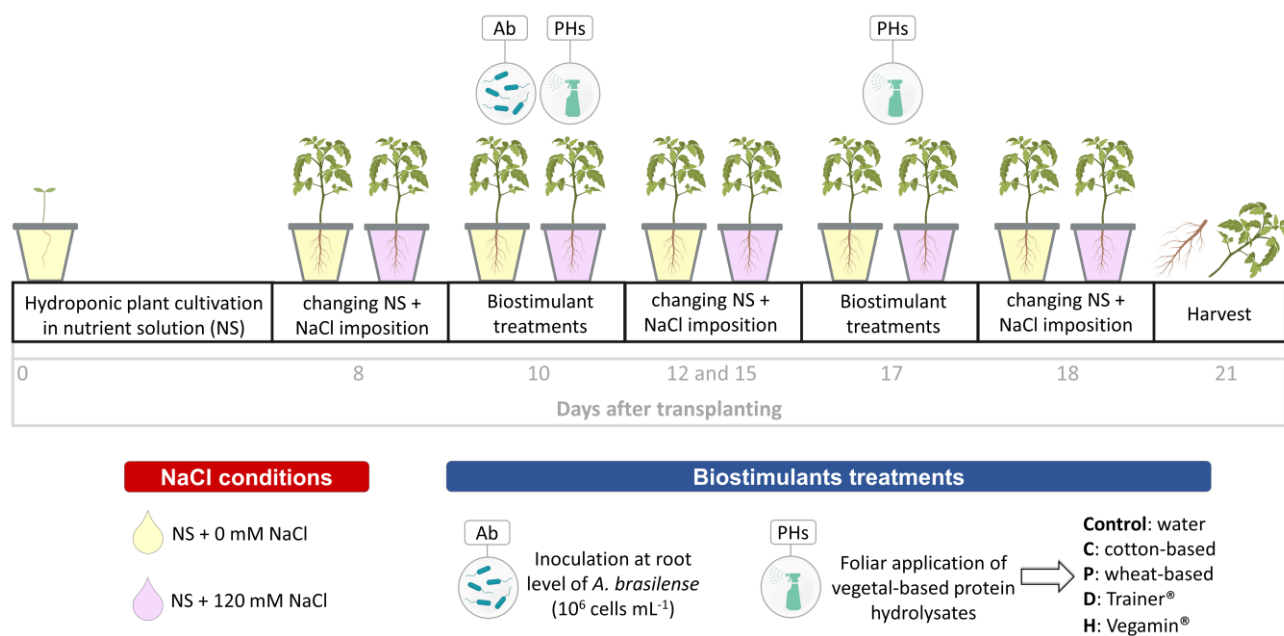

**Supplementary Figure S3.** Schematic representation of the experimental setup

Supplement: Supplementary file 1 [file ijms-23-14216-s001.zip › Supplementary Figure S3.pdf]
